# Apathy and fatigue, but not depression, associated with inflammatory biomarkers in older adults

**DOI:** 10.1002/alz.093242

**Published:** 2025-01-03

**Authors:** Fleur Harrison, Moyra E Mortby, Adam J Guastella, Julian N Trollor, Karen A Mather, Perminder S. Sachdev, Henry Brodaty

**Affiliations:** ^1^ Centre for Healthy Brain Ageing (CHeBA), University of New South Wales, UNSW Sydney, NSW Australia; ^2^ Neuroscience Research Australia, Sydney, NSW Australia; ^3^ UNSW Sydney, Sydney, NSW Australia; ^4^ UNSW Ageing Futures Institute, Sydney, NSW Australia; ^5^ University of Sydney, Sydney, NSW Australia; ^6^ Neuropsychiatric Institute, Prince of Wales Hospital, Randwick, NSW Australia

## Abstract

**Background:**

Apathy (loss of motivation or goal‐directed behaviour) and depression each confer risk for dementia, cardiovascular disease and mortality in older adults. Mechanisms for this are not yet understood, and may involve systemic inflammation. However, this has received little attention, particularly in older adults where depression may present differently. Symptoms of apathy, fatigue, and physical symptoms are more common in late‐life depression. This research aims to investigate whether apathy, depression and fatigue are differentially associated with inflammatory biomarkers in older adults.

**Method:**

1,037 community‐dwelling older adults without dementia (aged 70‐90, 55% women) completed self‐report assessments including measures of apathy and depression from the Geriatric Depression Scale, and fatigue from Assessment of Quality of Life‐6D. Inflammatory biomarkers from early morning fasting blood collection included C‐reactive protein (CRP) and interleukin‐6. Logistic regressions examined associations between levels of biomarkers and apathy, depression and fatigue separately. Analyses were initially unadjusted, then adjusted for baseline age, sex, education, global cognition (MMSE), health conditions, medications and BMI.

**Result:**

Interleukin‐6 was associated with apathy, depression and fatigue in unadjusted models (odds ratio [OR] per natural log unit increase in IL‐6: 1.73, 95% confidence interval [CI] 1.38‐2.22; OR 1.56, 95% CI 1.12‐2.15; OR 1.72, 95% CI 1.14‐2.59 respectively). The association with apathy remained significant after adjustment for other symptoms, socio‐demographics, cognition and health‐related covariates, but findings for depression and fatigue were attenuated. CRP was associated with apathy and fatigue (OR 1.10, 95% CI 1.00‐1.21; OR 1.34, 95% CI 1.11‐1.60 respectively) although the former was attenuated by adjustment for covariates. Previous associations between CRP and depression were not replicated.

**Conclusion:**

In older persons, apathy and fatigue were differentially linked with inflammatory biomarkers, whereas previous associations between inflammation and depression were not replicated. Findings confirm the importance of fatigue, and provide novel insight into apathy, as prevalent and impairing symptoms which map to peripheral inflammation in older adults. Inflammation may at least partially underly the complex associations between apathy, depression and poorer health outcomes including dementia.

